# TIGIT/CD226 Axis Regulates Anti-Tumor Immunity

**DOI:** 10.3390/ph14030200

**Published:** 2021-02-28

**Authors:** Jinah Yeo, Minkyung Ko, Dong-Hee Lee, Yoon Park, Hyung-seung Jin

**Affiliations:** 1 Center for Theragnosis, Biomedical Research Institute, Korea Institute of Science and Technology (KIST), Seoul 02792, Korea; jinahyeo@kist.re.kr (J.Y.); yuiopmk15@kist.re.kr (M.K.); 2 Department of Convergence Medicine, Asan Institute for Life Sciences, Asan Medical Center, University of Ulsan College of Medicine, Seoul 44610, Korea; nerd.is.high@gmail.com

**Keywords:** cancer immunotherapy, immune checkpoint blockade, CD226, TIGIT, PVR

## Abstract

Tumors escape immune surveillance by inducing various immunosuppressive pathways, including the activation of inhibitory receptors on tumor-infiltrating T cells. While monoclonal antibodies (mAbs) blocking programmed cell death 1 (PD-1), programmed death-ligand 1 (PD-L1), and cytotoxic T lymphocyte-associated antigen 4 (CTLA-4) have been approved for multiple cancer indications, only a subset of patients benefit from immune checkpoint blockade therapies, highlighting the need for additional approaches. Therefore, the identification of new target molecules acting in distinct or complementary pathways in monotherapy or combination therapy with PD-1/PD-L1 blockade is gaining immense interest. T cell immunoreceptor with Ig and immunoreceptor tyrosine-based inhibitory motif (ITIM) domains (TIGIT) has received considerable attention in cancer immunotherapy. Recently, anti-TIGIT mAb (tiragolumab) has demonstrated promising clinical efficacy in non-small cell lung cancer treatment when combined with an anti-PD-L1 drug (Tecentriq), leading to phase III trial initiation. TIGIT is expressed mainly on T and natural killer cells; it functions as an inhibitory checkpoint receptor, thereby limiting adaptive and innate immunity. CD226 competes for binding with the same ligands with TIGIT but delivers a positive stimulatory signal to the immune cells. This review discusses the recent discoveries regarding the roles of TIGIT and CD226 in immune cell function and their potential application in cancer immunotherapy.

## 1. Introduction

**Figure 1:**
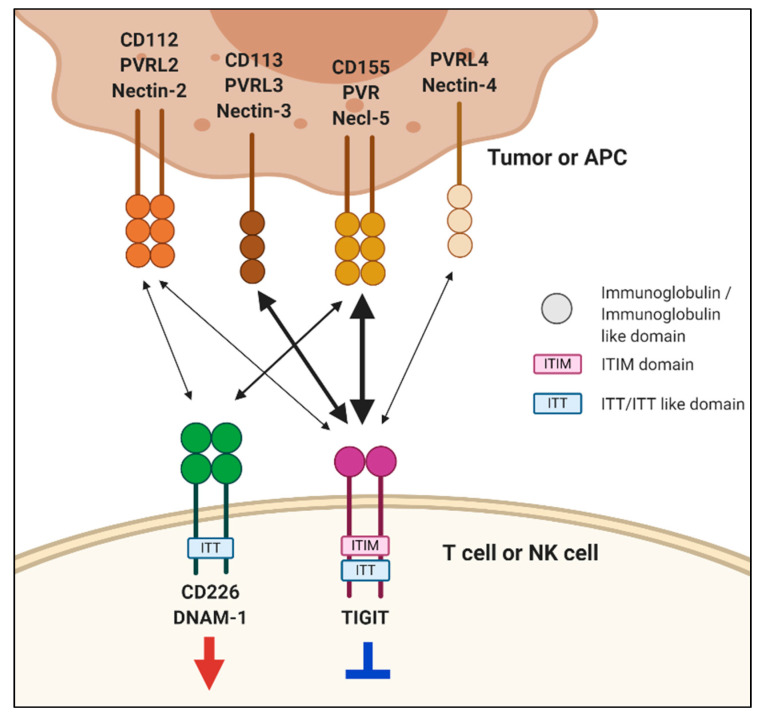
Interactions of T cell immunoreceptor with Ig and immunoreceptor tyrosine-based inhibitory motif (ITIM) domains (TIGIT) and CD226 with nectin and nectin-like molecules. TIGIT and CD226 are mainly expressed on T and natural killer (NK) cells. TIGIT has multiple ligands, including poliovirus receptor (PVR), nectin-2, nectin-3, and nectin-4. TIGIT binds to nectin-2 and nectin-3 with lower affinity than PVR. Upon engagement, TIGIT transmits inhibitory signals through ITIM and immunoglobulin tyrosine tail (ITT)-like motifs in its cytoplasmic domain. CD226 interacts with PVR and nectin-2 to deliver a positive signal. TIGIT binds to PVR with higher affinity than CD226. The integrated signals formed by their complex interactions regulate immune-cell functions, which is important for immunity and inflammatory responses. Interactions between receptors and ligands are depicted by two-sided arrows. The arrows are proportional to the reported affinities of the interactions except nectin-4.

Cancers harbor genetic alterations. The adaptive immune system discriminates between normal and cancer cells according to the protein products of these genetic alterations. T cells express T cell receptors (TCRs) that can recognize antigenic peptides presented by major histocompatibility complex (MHC) molecules [1,2]. Tumor antigens may trigger anti-tumor T cell responses; however, tumor-infiltrating lymphocytes (TILs) fail to efficiently eradicate the cancer cells. This is largely because TILs become dysfunctional or exhausted in the tumor environment, presumably due to persistent tumor antigen stimulation and the presence of immunosuppressive molecules. It is increasingly clear that exhausted T cells (T_ex_) are the major target population for immune checkpoint blockade (ICB) therapy [3,4]. T_ex_ cells exhibit distinct functional and phenotypic properties, such as impaired proliferation, decreased cytokine production, and high expression of co-inhibitory receptors including cytotoxic T lymphocyte-associated antigen-4 (CTLA-4, CD152), programmed death-1 (PD-1, CD279), T cell immunoglobulin and mucin-domain containing protein-3 (TIM-3, CD366), lymphocyte activation gene-3 (LAG-3, CD223), and T cell immunoreceptor with Ig and immunoreceptor tyrosine-based inhibitory motif (ITIM) domains (TIGIT) [5]. Tumor-derived ligands that interact with the co-inhibitory receptors, such as programmed death-ligand 1 (PD-L1), directly inhibit anti-tumor T cell responses, thereby promoting tumor immune escape [6]. Blockade of the PD-1/PD-L1 pathway using either anti-PD-1 or anti-PD-L1 monoclonal antibodies (mAbs) has only been successful in a subset of patients with particular cancer types [7,8]. Presently, numerous studies have been conducted to improve the therapeutic efficacy of PD-1/PD-L1 blockades [9,10,11,12]. TIGIT family receptors are a cluster of immunoglobulin superfamily receptors, which interact with nectin and nectin-like molecules (Necls) [13]. This group includes TIGIT, CD226 (also known as DNAX accessory molecule [DNAM]-1), CD96 (also known as T cell activation, increased late expression [TACTILE]), and CD112R (also known as poliovirus receptor-related immunoglobulin domain-containing [PVRIG]) [14]. Competitive or cooperative interactions between these receptors and their cognate ligands modulate immune cell activation (Figure 1) [15,16]. Among these, in the present study, we focus on the roles of TIGIT and CD226 in regulating T and natural killer (NK) cell function and the potential therapeutic application of these receptors in cancer immunotherapies.

## 2. TIGIT

### 2.1. TIGIT Structure and Its Ligands

TIGIT is a transmembrane glycoprotein comprising one immunoglobulin variable (IgV) domain, a type I transmembrane domain, and a cytoplasmic tail with an immunoreceptor tyrosine-based inhibitory motif (ITIM) and immunoglobulin tyrosine tail (ITT)-like motif [17,18,19]. The cytoplasmic tail of TIGIT initiates an inhibitory signaling cascade. Previous studies have reported that ITT-like motif (Tyrosine, Y225) mediates a major inhibitory signal in humans, whereas mouse TIGIT inhibitory signal can be triggered by either the ITIM (Y277) or the ITT-like motif residue (Y233) [20]. Upon binding to its ligand, the cytoplasmic tail of TIGIT is phosphorylated and binds to cytosolic adaptor growth factor receptor-bound protein 2 (Grb2), recruiting Src homology 2 (SH2)-containing inositol phosphate-1 (SHIP-1). SHIP-1 inhibits phosphoinositide 3 kinase (PI3K) and mitogen-activated protein kinase (MAPK) signaling cascades [21]. Moreover, phosphorylated TIGIT associates with beta-arrestin 2 and recruits SHIP-1, which further suppresses the auto-ubiquitination of tumor necrosis factor (TNF) receptor-associated factor 6 (TRAF-6) to inhibit nuclear factor kappa B (NF-κB) activation [21,22].

TIGIT has multiple ligands, including PVR (Necl-5 or CD155), nectin-2 (CD112), nectin-3 (CD113), and nectin-4 (PVRL4) [13,23]. Nectin and Necl proteins are cell-surface glycoproteins that belong to the immunoglobulin superfamily. Nectin family comprises four members (nectin-1–4), and the Necl family consists of five members (Necl-1–5). They have three Ig ectodomains, which form homodimeric or heterodimeric complexes in the membrane [24]. The IgV domain of TIGIT exhibits sequence homology with PVR, nectin-1, nectin-2, nectin-3, and nectin-4 [17]. TIGIT binds to PVR with high affinity and nectin-2 and -3 with low affinity. Recently, nectin-4 has been reported to bind to TIGIT with an affinity similar to that of TIGIT and PVR binding [25]. PVR plays immunoregulatory roles by interacting with TIGIT, CD226, and CD96 [26,27,28]. PVR has a greater affinity for TIGIT than either CD226 or CD96, implying a dominant role of TIGIT inhibitory signaling over activation signals. Furthermore, PVR expression is commonly upregulated in several types of cancer and tumor-associated myeloid cells [29,30]. Elevated PVR expression has been associated with an unfavorable prognosis across various solid cancer types [31,32]. Nectin-2 interacts with TIGIT, CD226, and CD112R; however, both TIGIT and CD226 have much weaker binding affinity to nectin-2 than PVR. Similar to PVR, the TIGIT–nectin-2 interaction could transduce an inhibitory signal, but the CD226–nectin-2 interaction triggers immune cell activation. A recent study has demonstrated that the inhibitory effect of nectin-2 is mediated by CD112R and not TIGIT [14].

### 2.2. Role of TIGIT in Immune Cell Regulation

TIGIT is expressed on most NK and multiple T cell subsets, including memory and activated T cells, regulatory T cells (T_reg_), and follicular T helper cells (T_FH_) [17,19,20]. Upon activation with its ligands, TIGIT expression is upregulated in both T and NK cells, where TIGIT inhibits cytotoxic activity. TIGIT-deficient mice do not develop spontaneous autoimmunity; however, they exacerbate experimental autoimmune encephalitis when immunized with myelin oligodendrocyte glycoprotein, indicating a suppressive role of TIGIT [27]. In preclinical mouse tumor models, TIGIT deficiency delays the subcutaneous growth of both B16F10 and MC38 cells and lung metastasis of B16 cells [33,34]. Moreover, TIGIT-deficient mice show increased survival when challenged with VK*MYC myeloma cell lines [35]; however, a recent study revealed that TIGIT-deficient mice did not reject the implanted B16F10 and MC38 more efficiently compared with wild-type (WT) mice [36]. Moreover, in B16F10, RM-1, and E0771 cell lung metastasis models, the beneficial effect of TIGIT deficiency on tumor metastasis was not observed [37,38]. These discrepancies might be results of different experimental setups and/or mouse housing conditions [39]. Further studies with immune cell-type-specific TIGIT-deficient mouse models would be helpful to clarify the suppressive role of TIGIT in vivo [34].

Several mechanisms may explain TIGIT-mediated inhibition of T and NK cell activities. First, as aforementioned, TIGIT delivers an inhibitory signal resulting from ITIM and/or ITT motifs within its cytoplasmic domain. Agonistic anti-TIGIT antibodies inhibit human and mouse T cell proliferation and cytokine production without antigen presenting cells (APC) by suppressing T cell receptor/CD28-activating signaling [27,40]; however, TIGIT engagement increases the expression of receptors for T cell maintenance (e.g., interleukin [IL]-2R, IL-7R, and IL-15R) and anti-apoptotic molecules (e.g., Bcl-xL) [27], implying that TIGIT signaling could mediate the survival of T_ex_ cells. Additionally, TIGIT signaling also inhibits cytotoxicity, degranulation, and cytokine secretion of NK cells [19,41]. Moreover, TIGIT disrupts CD226 co-stimulation. TIGIT has higher affinity for the same set of ligands (PVR and CD112) than CD226. Thus, TIGIT outcompetes CD226 for binding to its ligands [17]. Knockdown of TIGIT in human CD4^+^T cells induces T-bet-mediated interferon (IFN)-γ production, which can be overcome by blocking CD226-CD155 signaling [40]. Additionally, TIGIT hinders CD226 signaling through the physical prevention of CD226 homodimerization [42]. A recent study by Jin et al. has demonstrated that TIGIT directly affects the intracellular regulation of CD226 activation. By using an antibody specifically recognizing the phosphorylated form of CD226 (phospho-Y322), they have shown that CD226 phosphorylation at Y322 is reduced in TIGIT WT-expressing Jurkat cells upon PVR engagement but not in the cells expressing TIGIT mutant (Y225A/Y231A) [43]. In addition, TIGIT has been known to suppress T cell function in a cell-extrinsic manner. Following TIGIT ligation, PVR signaling leads to increased production of IL-10 and diminished production of IL-12p40 in human dendritic cells (DCs), which further downregulates T cell activation [17]. In accordance with this result, TIGIT ligation inhibits macrophage activation and leads to increased M2 macrophage polarization through PVR [44].

The role of TIGIT has been implicated in modulating T_reg_ cell responses. [45,46]. TIGIT expression is observed in a subset of natural T_reg_ cells in both mice and human. TIGIT^+^T_reg_ cells express higher levels of T_reg_ signature genes, including *Foxp3*, *CD25*, and *CTLA-4*, compared with TIGIT^−^T_reg_ cells. TIGIT expression is strongly correlated with the suppressive capacity and the lineage stability of human T_reg_ cells [45,46,47]. Furthermore, TIGIT engagement leads to the induction of IL-10 and fibrinogen-like protein 2, which selectively suppress T helper type 1 (Th1) and Th17 responses [45].

### 2.3. Targeting TIGIT for Cancer Immunotherapy

#### 2.3.1. TIGIT as a Potential Prognostic Marker for Cancer

Accumulating data from the immune monitoring of cancer patients have revealed that TIGIT expression is elevated in T and NK cells, and it often appears to be associated with advanced disease status and poor clinical outcomes [34,35,48,49,50,51,52,53,54,55,56,57,58]. In follicular lymphoma (FL) patients, TIGIT is highly expressed on intratumoral T_reg_ and late-stage memory CD8^+^T cells, and increased numbers of TIGIT-expressing tumor infiltrating T cells reveal a correlation with poor survival rate [48]. Multidimensional flow cytometric analysis of intratumoral T cells obtained from FL patients before and after anti-PD-1 therapy has revealed that TIGIT^+^ T_ex_ cells majorly respond to this therapy. It has been observed that TIGIT^+^ exhausted T cell populations are downregulated and TIGIT^+^ effector cells are upregulated by anti-PD-1 therapy [48]. Increase in the proportion of highly suppressive tumor-infiltrating T_reg_ cells following TIGIT expression is associated with poor clinical outcomes in patients with hepatocellular carcinoma (HCC) and metastatic melanoma [47,57]. Moreover, upregulation of TIGIT indicates unfavorable disease status. High-risk patients with myelodysplastic syndrome (MDS) express higher levels of TIGIT and PD-1 in peripheral blood T and NK cells than low-risk patients [58]. High TIGIT expression renders CD4^+^T, CD8^+^T, and NK cells hypo-responsive to stimulation in high-risk MDS patients. Several studies have reported that TIGIT upregulation after treatment is correlated with recurrence. In patients with high-grade serous carcinoma, NanoString analysis of tumor tissues has indicated that recurrent tumors acquire a more inflamed phenotype with increased expression of TIGIT, CTLA4, Lag-3, and Tim-3 compared to primary tumors [59]. The proportion of TIGIT^+^CD8^+^T cells is increased in peripheral blood collected from acute myeloid leukemia (AML) patients, and it becomes more evident in patients with primary refractory disease and leukemia relapse post-allogeneic stem-cell transplantation [49]. Furthermore, TIGIT and/or PD-1 expression in CD8^+^T cells is increased in patients with gastric cancer relapse after treatment with SOX (S-1 and oxaliplatin) regimen, whereas no notable increase in the proportion of TIGIT^+^ and/or PD-1^+^CD8^+^T cells was found in relapse-free patients [60]. The compensatory increase in TIGIT expression post-treatment has also been observed in high-grade neuroendocrine neoplasms upon anti-PD-1 therapy [61].

#### 2.3.2. TIGIT Blockade in Anti-Tumor Immunity

Based on the mechanism underlying TIGIT-mediated regulation of anti-tumor immune responses, efforts have been made to enhance T or NK cell activity by blocking TIGIT binding to its ligands, PVR and nectin-2, with monoclonal antibodies (mAbs) for therapeutic interventions. Several preclinical mouse models have been used to assess the anti-tumor efficacy of anti-TIGIT blocking mAbs. In CT26 colon carcinoma, EMT6 breast carcinoma, MC38 colon carcinoma, and GL261 glioblastoma models, treatment with anti-TIGIT-blocking mAbs combined with anti-PD-1 or PD-L1-blocking mAbs leads to nearly complete remission of tumor growth, whereas the treatment of anti-TIGIT mAbs as a single agent presents limited efficacy [42,62,63]. CD8^+^T cell depletion using anti-CD8α-depleting mAbs in CT26-bearing mice has revealed that the synergistic effect of dual blockade of TIGIT and PD-1 is mainly driven by the promotion of CD8^+^T cell responses. A triple combination of anti-TIGIT mAbs, anti-PD-L1 mAbs, and radiotherapy elicits almost complete remission of tumor growth in CT26-bearing mice [64].

Sufficient tumor regression by treatment with anti-TIGIT mAbs alone has been reported in different mouse tumor models. In multiple myeloma (MM) mouse tumor model, TIGIT blockade leads to reduced tumor growth and increased survival compared with mice receiving control IgG or anti-PD-1 mAbs [35]. Moreover, TIGIT blockade presents anti-tumor efficacy in *Tgfbr1/Pten2* conditional knock-out (KO) mouse model that spontaneously develops head and neck squamous cell carcinoma (HNSCC) upon tamoxifen injection [55,65]. Both studies suggest that TIGIT is highly expressed on CD8^+^T and T_reg_ cells in MM or HNSCC TILs and that anti-TIGIT mAbs reverse TIGIT-mediated suppression of CD8^+^T cell effector functions; however, the potent anti-tumor effect of anti-TIGIT mAbs as a single agent may not be fully guaranteed simply by the increased expression of TIGIT in TILs, since high TIGIT expression is also observed in CD8^+^ TILs in CT26-bearing mice that are not responsive to TIGIT blockade [42]. A recent study by Chiu et al. provided additional insights into the mechanism through which TIGIT blockade mitigates tumor immune evasion and resistance to PD-1 blockade [66]. They found that anti-PD-1 mAb treatment induced the upregulation of TIGIT in CD8^+^ TILs in *Trp53* KO/*C-Myc*^OE^ mice, which is a highly aggressive HCC model; however, the compensatory expression of TIGIT upon PD-1 blockade was not observed in Hepa1-6-bearing mice that are known to be an anti-PD-1-sensitive orthotopic HCC model. PVRL1, which does not directly bind to TIGIT, contributed to TIGIT-mediated suppression of CD8^+^T cells by stabilizing PVR in HCC cells, and PVRL1 deficiency rendered HCC to be more sensitive to anti-PD-1 mAb treatment. In accordance with this finding, a differential level of the ligand expression, such as PVR and PD-L1, or an increase in the binding affinity of TIGIT to PVR under an acidic tumor microenvironment has been recently identified to contribute toward the sensitivity of tumor cells to TIGIT blockade [67,68].

Although TIGIT blockade is known to mainly act on CD8^+^T and T_reg_ cells, NK cell dependent efficacy of anti-TIGIT mAbs is also suggested. A recent study by Zhang et al. reported that treatment with anti-TIGIT mAbs 3 days after tumor cell implantation prevented tumor-infiltrating NK cell exhaustion in CT26 or methylcholanthrene (MCA)-induced fibrocarcinoma-bearing mice, which resulted in the enhancement of CD8^+^T cell responses and tumor rejection [34]. However, the mechanism through which TIGIT blockade has an impact primarily on NK cells compared to T cells and the mechanism through which NK cells promote CD8^+^T cell responses need to be further elucidated, since these results are contradictory to those of previous studies, revealing the CD8^+^T or the T_reg_ cell-mediated effect of TIGIT blockade using temporary depletion of these populations with specific antibodies [35,42,55]. A more recent study reported that anti-TIGIT mAbs enhanced IL-15-driven NK cell cytotoxicity in both B16F10 and LWT1 metastatic melanoma-bearing mice [69].

The potency of human anti-TIGIT blocking mAbs on CD8^+^T cells has been demonstrated in cancer patients. Cancer testis antigen NY-ESO-1-specific CD8^+^T cell responses are increased by the addition of blocking mAbs against TIGIT and/or PD-1 when peripheral blood mononuclear cells (PBMCs) from melanoma patients are stimulated with NY-ESO-1^157–165^ peptide. Furthermore, TIGIT blockade increases the capacity for proliferation and degranulation of CD8^+^TILs from advanced melanoma patients upon TCR stimulation using autologous non-CD3 cells and anti-CD3 mAbs [50]. Upon TCR stimulation with anti-CD2/anti-CD3/anti-CD28 microbeads, bone marrow (BM) CD8^+^T cells in MM patients show increased CD107a expression and cytokine production in response to TIGIT blockade [35]. When anti-TIGIT mAbs are added to ex vivo co-culture of CD3^+^TILs and Mel-624 cells expressing membrane-bound anti-CD3 scFv (Mel-624 OKT3), IFN-γ and IL-2 production by CD3^+^TILs from patients with endometrial, ovarian, kidney, head and neck, and lung cancers is promoted [14]. A recent study reported that antigen specific responses to CEF (CMV, EBV, flu) peptide are augmented by TIGIT blockade in peripheral blood CD8^+^T cells derived from pancreatic ductal adenocarcinoma (PDAC) patients after mFOLFIRINOX therapy [43].

#### 2.3.3. Mode of Action of Anti-TIGIT Therapy

Competitive binding of TIGIT and CD226 to PVR has been known as a key mechanism of TIGIT-driven immune suppression, and anti-TIGIT blocking mAbs are presumed to reverse the suppression by inhibiting TIGIT binding to PVR. This may occur as a mode of action; however, several questions need to be addressed for its clinical success and further translation of other members of TIGIT family receptors into cancer immunotherapy.

- Intracellular Regulation by Anti-TIGIT mAbs

Despite the importance of understanding the molecular interplay between TIGIT, CD226, and PVR, the mechanism through which extracellular signals from the receptor-ligand binding/receptor dynamics are integrated into the intracellular regulation, particularly in the context of anti-TIGIT therapy, remains unclear.

**Figure 2:**
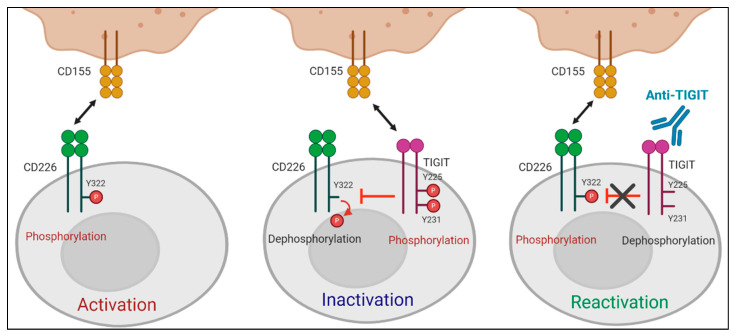
Role of CD226 in anti-TIGIT immunotherapy. TIGIT has a direct effect on intracellular regulation of CD226 activation in response to PVR binding. (**Left**) When TIGIT expression is absent or low, engagement of CD226 with PVR induces the phosphorylation of tyrosine 322 (Y322) on CD226, which leads to the activation of intracellular signaling cascades. (**Middle**) PVR preferentially binds to upregulated TIGIT over CD226. Upon interaction with PVR, the cytoplasmic tail of TIGIT is phosphorylated. This PVR-induced TIGIT phosphorylation inhibits T cell responses by promoting CD226 dephosphorylation. (**Right**) TIGIT blockade suppresses PVR-induced TIGIT phosphorylation and restores the impaired Y322 phosphorylation of CD226, thereby leading to T cell activation.

A recent study by Jin et al. reported that the effect of TIGIT blockade depends on tyrosine phosphorylation at Y322 of CD226, which was the first study to define the molecular requirements for anti-TIGIT blocking mAbs [43]. They showed that TIGIT-mediated intracellular inhibition of CD226 phosphorylation at Y322 was restored by TIGIT blockade. Moreover, CD226 mutant at Y322 (CD226^Y322A^) expressing CD8^+^T cells did not respond to TIGIT blockade, whereas CD226^WT^ or CD226^Y329A^ expressing CD8^+^T cells produced increased IFN-γ upon treatment with anti-TIGIT mAbs, suggesting that TIGIT blockade promotes T cell activation via CD226 phosphorylation at Y322 (Figure 2). CD226 dependent effect of anti-TIGIT mAbs was further shown in effector memory CD8^+^T cells expressing a low level of CD226 (CD226^lo^CD8^+^T_em_) not responsive to both antigen stimulation and anti-TIGIT mAb treatment. CD226 activation using anti-CD226 agonist mAbs renders CD226^lo^CD8^+^T_em_ responsive to TIGIT blockade.

- Isotype Selection of Anti-TIGIT mAbs

Recently, several studies have highlighted the importance of selecting appropriate fragment crystallizable (Fc) region for therapeutic antibodies. To date, the approved human therapeutic IgG antibodies belong to IgG1, IgG2, or IgG4 subclasses [70]. It is increasingly clear that binding of the Fc region of antibody to Fc gamma receptors (FcγRs) can elicit various immunomodulatory functions, including antibody dependent-cellular cytotoxicity (ADCC), complement-dependent cytotoxicity (CDC), and antibody-dependent cellular phagocytosis (ADCP) [71]. In addition, FcγR binding was reported to enhance agonistic activity of mAbs targeting tumor necrosis factor receptor superfamily members, such as CD28, CD137, CD40, and OX40 (CD134) [72].

**Figure 3:**
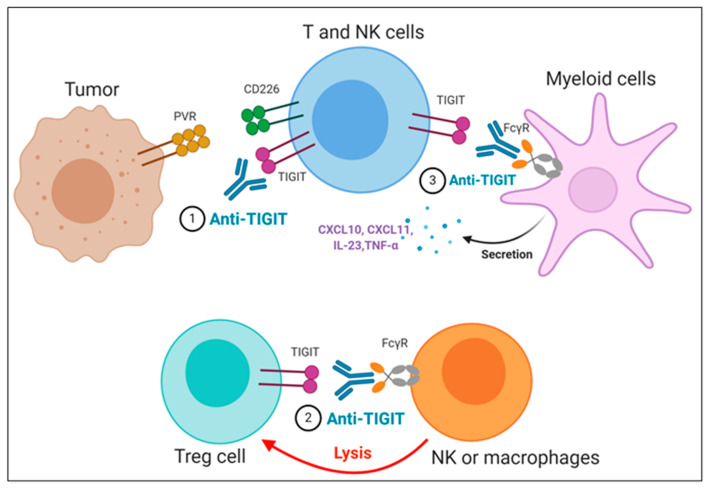
Proposed mechanisms of anti-TIGIT monoclonal antibodies (mAbs) in cancer immunotherapy. (**1**) Blockade of TIGIT could reverse the exhaustion of T and NK cell-mediated anti-tumor immunity. (**2**) Intratumoral regulatory T cells (T_reg__) cells expressing high levels of TIGIT could be preferentially depleted by anti-TIGIT mAbs, presumably through antibody-dependent cellular phagocytosis (ADCP) by macrophages and/or antibody-dependent cellular cytotoxicity (ADCC) by NK cells. (**3**) The TIGIT mAb- fragment crystallizable (Fc) gamma receptors (FcγR) engagement could activate myeloid cells, leading to enhanced antigen presentation function and proinflammatory chemokine and cytokine secretion.

The importance of the Fc domain of anti-TIGIT mAb is emphasized by the findings that anti-TIGIT mAb with Fc devoid of effector functions, which was intended to solely block TIGIT binding to its ligands, fails to exert any of anti-tumor efficacies in preclinical models [36,73,74]. It may be due to the loss of its depleting activity against TIGIT-expressing intratumoral T_reg_ cells, which has been considered as a potential mechanism of anti-TIGIT mAb-mediated anti-tumor effect [74]; however, it is still not clear whether the anti-tumor efficacy of anti-TIGIT mAbs depends on T_reg_ depletion, since there are recent reports that anti-TIGIT mAbs on mIgG2a isotype induce anti-tumor responses without evidence of T_reg_ depletion in mouse tumor models [36,73]. It may possible that FcγR on APC could act as a scaffold to crosslink anti-TIGIT mAb bound to TIGIT on immune cells, which may enhance the effect of TIGIT antagonism independent of T_reg_ cells. In addition, Han et al. recently have shown that the antibody-FcγR engagement induced activation of myeloid cells, leading to pro-inflammatory chemokine and cytokine secretion (Figure 3) [36]. Comparison of clinical activities of anti-human TIGIT mAbs with different Fc scaffolds could provide insight into whether FcγR binding is required for optimal anti-tumor responses of TIGIT blockades.

### 2.4. Anti-TIGIT Antibodies in Clinical Trials

Approximately 10 human anti-TIGIT mAbs, which have different IgG isotypes or mutant forms, have entered clinical trials. Table 1 summarizes publicly available data regarding antibody isotype, combination with different drugs, current development phase, and cancer types. Numerous clinical trials are evaluating the safety and the efficacy of anti-TIGIT mAb either as a monotherapy or in combination with PD-1/PD-L1 blockade or chemotherapies for the treatment of various cancers. Recently, the phase II CITYSCAPE trial presented significant response rates of tiragolumab plus atezolizumab in PD-L1-positive non-small cell lung cancer (NSCLC). The study revealed a significant objective response rate (ORR) improvement for the combination group (37% vs. 21%) as well as progression-free survival (PFS) improvement (5.6 vs. 3.9 months; hazard ratio [HR] 0.58). Importantly, patients in the combination group with high PD-L1 expression had an ORR of 66% compared with 24% in the atezolizumab group [75]. 

## 3. CD226

### 3.1. CD226 Structure and Its Ligands

CD226 is widely expressed in immune cells including T cells, NK cells, and monocytes [76]. CD226 is a transmembrane glycoprotein that comprises two immunoglobulin V-like domains (D1 and D2), a type I transmembrane domain, and an intracellular domain. The intracellular domain harbors two highly conserved phosphorylation sites, Y322 and S329, in humans (Y319 and S326 in murine ortholog). CD226 interacts with PVR and nectin-2. A structural analysis has revealed that the extracellular D1 domain of CD226 binds to PVR via a conserved docking mode [77]. Whether the D2 domain of CD226 is critical for its binding to PVR needs to be further investigated [78]. The measured solution binding affinity between human CD226-Fc and PVR-Fc is similar to that between CD226-Fc and nectin-2-Fc; however, CD226-Fc binds less efficiently to nectin-2 than PVR-expressing cells, suggesting that the homophilic interaction of nectin-2 might hinder CD226 binding to nectin-2 [79]. In addition, it has been reported that mouse CD226 only interacts with mouse PVR but not mouse nectin-2, which needs further clarification. Both PVR and nectin-2 expressions are upregulated on tumor cells, which contributes to tumor recognition and killing. Indeed, loss of PVR and nectin-2 on acute myeloid leukemia cells renders them resistant to NK cell-mediated killing [80]

PVR can be expressed in soluble form, lacking the transmembrane region, by alternative splicing in humans. Moreover, the mechanism by which the expression of membrane-bound PVR and soluble PVR (sPVR) is regulated remains unclear. High levels of sPVR are observed in the serum samples of patients with various types of cancers [81,82]. Okumura et al. have reported that sPVR inhibits CD226-mediated cytotoxicity of NK cells in a mouse tumor model. They suggested that sPVR could compete with membrane bound PVR and function as a neutralizing molecule for CD226 in NK cells. Interestingly, sPVR bound preferentially to CD226 over TIGIT and CD96, implying monomeric and dimeric PVR may function differently [83].

### 3.2. CD226 Signaling

The CD226 signaling pathway has been widely studied in NK cells. Upon engagement through corresponding ligands, CD226 is localized to lipid rafts and binds to the actin cytoskeleton through its association with human disc large protein or synapse-associated protein 97 (SAP97), the membrane-associated guanylate kinase homolog (MAGUK), and the actin-binding protein 4.1G [84,85]. During the formation of immunological synapse, CD226 transmits an activating signal, and thereafter, it induces the aggregation of lymphocyte function-associated antigen 1 (LFA-1) [86]. Protein kinase C (PKC) phosphorylates the S326 residue of CD226. This causes the association of LFA-1 with CD226. LFA-1 binds to intercellular adhesion molecule 1 (ICAM-1) and promotes its conformational change, leading to the recruitment of Fyn that phosphorylates the Y319 residue of CD226 [86]. CD226 phosphorylation at Y319 triggers activation of extracellular signal-regulated kinase (ERK) and AKT in NK cells upon the engagement of CD226 by the agonist mAbs, which is critical for NK cell cytotoxicity [87]. The physiological importance of CD226 Y319 phosphorylation is assessed in *CD226^Y319F^* KI (knock-in) mice that exhibit impaired cytotoxicity and cytokine production by NK cells. A similar observation on the role of CD226 phosphorylation at Y322 is made in human CD8^+^T cells. Exogenous expression of CD226^WT^ or CD226^Y322A^ in human CD8^+^T cells revealed that PVR-induced CD226 phosphorylation at Y322 is required for downstream signaling activation including ERK, p38, and AKT and corresponding T cell responses [43]. Next, the CD226 downstream signaling cascade leads to the phosphorylation of lymphocyte cytosolic protein 2 (LCP2) and vav guanine nucleotide exchange factor 1 (Vav1) [88]. Moreover, it activates phosphatidylinositol-4,5-bisphosphate phosphodiesterase gamma-2 (PLCγ2), ERK, and AKT downstream, thereby allowing degranulation and calcium mobilization [89]. The activated AKT phosphorylates forkhead box protein O1 (FOXO1) transcription factor, which induces the translocation of FOXO1 from the nucleus to the cytoplasm, where it is degraded and inactivated, thereby removing the negative regulator of NK cell activation [90].

### 3.3. CD226 in Tumor Immunity

The importance of the CD226–PVR axis in regulating tumor immunity has been shown in vitro and in vivo in preclinical mouse models. CD8^+^ T cells or DX5^+^ (CD49b) NK cells isolated from CD226-deficient mice are less cytotoxic to PVR-expressing tumor cells but not to PVR-negative tumor cells [91]. Moreover, reduced proliferative capacity of CD226 deficient-OT-I CD8^+^T cells was observed upon stimulation with the ovalbumin (OVA) peptide pulsed EL4 cells expressing PVR. However, the proliferation of CD226-deficient OT-I T cells was not impaired when stimulated with OVA peptide pulsed dendritic cells, suggesting that CD226 may promote effector T cell function in environments where co-stimulatory ligand expressions is limited, such as in tumors [92]. Consistent with the in vitro results, CD226 deficient mice also display a greater tumor burden than WT mice to a variety of tumors [38,90,92,93]. Impaired NK-cell-mediated suppression of tumor growth by CD226 deficiency has been reported in B16/F10 or RM-1 lung-metastases mouse models [38,92]. The effect of inhibiting the CD226–PVR axis on anti-tumor immune responses was investigated using anti-CD226 blocking mAbs. Blockade of CD226 with anti-CD226 antagonist mAb did not influence the tumor growth in mice [42,94,95]. However, administering anti-CD226 mAbs to mice treated with the combination of anti-TIGIT and anti-PD-L1 mAbs or anti-PD-1 and anti-GITR (glucocorticoid-induced TNFR-related protein) mAbs reversed the anti-tumor effect and the survival benefit of the combined treatment, which was accompanied by reduced effector function and frequency of CD8^+^T cells at the tumor site [42,95].

#### 3.3.1. CD226 Downregulation in Dysfunctional T Cells

CD226 downregulation has been reported in T or NK cells of patients with cancer or human immunodeficiency virus (HIV) [26,43,49,50,96,97,98,99,100,101,102,103], which most likely occurs with an upregulation of PD-1 and TIGIT and impaired functionality. NY-ESO-1-specific CD8^+^TILs express low levels of CD226 and high levels of TIGIT and PD-1 in melanoma patients, but this imbalance is not found in circulating CD8^+^T cells regardless of specificity for NY-ESO-1 [50]. Moreover, CD226 expression is inversely proportional to TIGIT expression in peripheral blood CD8^+^T cells from AML patients [49]. A similar phenotype is observed in CD8^+^TILs from patients with renal cell carcinoma (RCC), colorectal cancer (CRC), and NSCLC that display upregulation of PD-1, TIGIT, Lag-3, and Tim-3 with reduced CD226 expression [43]. Further phenotypic dissection of the CD8^+^TILs from mouse tumor models reveals that an exhausted phenotype is presented with an increased expression of TIGIT, PD-1, Tim-3, Lag-3, CD101, CD38, and eomesodermin (Eomes) in CD226^lo^CD8^+^TILs. Consistent with the phenotypic features, both polyfunctionality and proliferative capacity are attenuated in CD226^lo^CD8^+^TILs compared with CD226^hi^CD8^+^TILs [43]. The correlation between CD226 downregulation and functional defect of CD8^+^T cells is presented in a mouse MM model that had experienced a relapse after autologous stem cell transplantation. BM CD8^+^T cells from relapsed MM mice display phenotypic and functional characteristics of exhaustion together with reduced CD226 expression, whereas MM-controlled mice retain high CD226 expression in the BM [102]. A recent study reported the transcriptional differences between CD226^hi^ and CD226^neg^ CD8^+^TILs using single-cell RNA sequencing in conjunction with cellular indexing of transcriptomes and epitopes by sequencing [99]. Furthermore, gene enrichment related to effector function and immunological synapse formation was found in CD226^hi^CD8^+^TILs from HCmel12^hgp100^ melanoma-bearing mice; however, unlike the previous studies on the inverse correlation between CD226 and co-inhibitory receptor expression on the surface of CD8^+^TILs, the expression of co-inhibitory receptor genes was unaltered in CD8^+^TILs regardless of CD226 expression. This discrepancy may occur from heterogeneity or different status of T_ex_ cell differentiation depending on the tumor burden or variations in tumor models. Indeed, the gene expression profiles in CD226^neg^CD8^+^TILs did not appear to be uniformly defined as a particular subset [99].

A similar approach was employed in human CD8^+^T cells from healthy donors to delineate the molecular differences between CD226^+^ and CD226^−^ CD8^+^T cells under resting state and upon TCR stimulation [100]. Resting CD226^−^ CD8^+^T_em_ cells displayed gene expression profiles comparable to CD226^+^CD8^+^T cells. Although genes involved in T cell activation were found in both CD226^−^ and CD226^+^CD8^+^T_em_ cells upon TCR stimulation, activated CD226^−^ CD8^+^T_em_ cells revealed the enrichment of gene signatures of resting T cells, T_reg_ cells, and TGF-β signaling, which would contribute toward understanding the hypo-responsiveness of CD226^−^CD8^+^T_em_ cells upon TCR/CD28 or antigen-specific stimulation [43,100]; however, it remains unclear whether the gene profiles in CD226^−^CD8^+^T_em_ cells are the cause or the result of CD226 downregulation. Since the association of CD226 downregulation with progressive differentiation has been demonstrated in human CD8^+^T cells under steady-state conditions [43] and upon aging [103], further genetic and epigenetic insights are required to decipher the role of CD226 in T cell regulation. CD226 downregulation is also found in T_reg_ cells, γδ T cells, and NK cells of cancer patients. The proportion of highly suppressive CD25^hi^Foxp3^+^T_reg_ cells is increased in melanoma patients who present a high TIGIT/CD226 ratio in tumor-infiltrating T_reg_ cells. This ratio is also associated with dismal clinical outcome after anti-PD-1 and/or anti-CTLA4 therapies [47]. Increased γδ T cell proportions with high TIGIT and low CD226 expression are correlated with decreased overall survival rates in AML patients [101]. In addition, the inverse expression between CD226 and TIGIT and/or PD-1 is indicative of the disease status in MDS patients [58].

#### 3.3.2. Mechanisms of CD226 Downregulation

**Figure 4:**
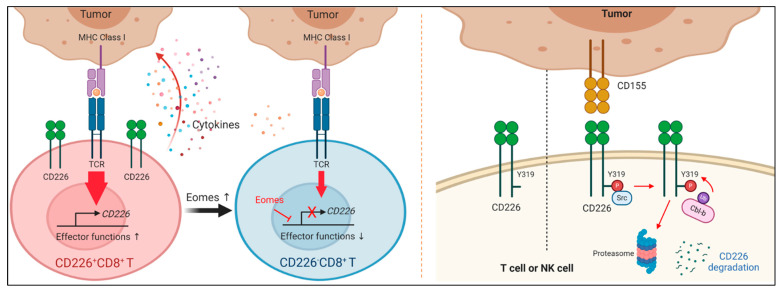
Mechanisms of CD226 downregulation. (**Left**) Tumor microenvironment promotes the accumulation of a subset of CD8^+^ T cells that lose CD226. CD226 is transcriptionally downregulated in an eomesodermin (Eomes)-dependent and a PVR-independent manner. CD226 downregulation is abolished in Eomes-deficient CD8^+^ T cells. Eomes directly interacts with regulatory elements of the *CD226* gene. (**Right**) CD226 expression is posttranslationally regulated through the ubiquitin–proteasome pathway. After engagement with PVR, mouse CD226 is phosphorylated at Y319 by Scr kinase, subsequently recruiting E3 ubiquitin ligase Cbl-b, which induces ubiquitination-dependent proteasomal degradation of phosphorylated CD226.

Recent studies have suggested that the downregulation of CD226 is mediated via both an eomesodermin (Eomes)-dependent transcriptional mechanism and a CD155-mediated posttranslational mechanism (Figure 4) [99,100]. Weulersse et al. found that Eomes level is higher in CD226^−^CD8^+^ T cells and that CD226 downregulation is abrogated in Eomes-deficient CD8^+^ T cells. In contrast, CD226^−^ CD8^+^ T cells are increased in the spleens of Eomes-overexpressing mice. Eomes is recruited to a regulatory region of *CD226*, suggesting it may directly regulate CD226 expression at the transcriptional level; however, since Eomes is a well-known key transcription factor for modulating homeostasis of both CD8^+^memory T and T_ex_ cells [4], it needs to be further addressed whether Eomes directly regulates CD226, or affected immune responses by Eomes upregulation is responsible for CD226 downregulation. Indeed, all Eomes-expressing T cells do not lose CD226 expression, suggesting that there are other factors regulating CD226 transcription [99]. Braun et al. showed that CD226 expression is higher on TILs in CD155 deficient tumors than in the WT tumors. Mice with a CD226^Y319F^ mutation have increased frequencies of CD226^hi^CD8^+^ TILs, correlating with enhanced effector function against tumors. Engagement with PVR induces decreased surface expression of CD226, which is dependent on CD226 Y319 phosphorylation. E3 ubiquitin ligase Casitas B-lineage lymphoma proto-oncogene-b (Cbl-b) could be involved in ubiquitination-dependent degradation of CD226 [100]. However, further clarification on the role of CD226 phosphorylation at Y319 or Y322 in regulating T cell activation is required since these results suggest a conflicting role to those of previous studies [43,87].

#### 3.3.3. Predictive Value of CD226 for Immune Checkpoint Blockade Therapy

In accordance with the CD226-dependent regulation of CD8^+^T cell response, the differential response between CD226^hi^ and CD226^lo^CD8^+^T cells is reported in immune checkpoint blockade therapies, including anti-TIGIT or anti-PD-1 mAbs. Upon stimulation with CEF peptide, FACS-sorted CD226^lo^CD8^+^T_em_ cells from healthy donors fail to respond to TIGIT and/or PD-1 blockade, whereas the CEF-specific responses of CD226^hi^CD8^+^T_em_ cells are enhanced [43]. This observation is further validated in a translational setting. Upregulation of CD226 is found in peripheral blood CD8^+^T cells from PDAC patients after mFOLFIRINOX chemotherapy, which is associated with an elevated responsiveness of antigen-specific CD8^+^T cells to treatment with anti-TIGIT or anti-PD-1 mAbs, and has also been reported in the mouse tumor models [43]. Wang et al. demonstrated that combination treatment of anti-PD-1 mAbs with anti-GITR agonist mAbs elicited anti-tumor efficacy in MC38-bearing mice in a CD226-dependent manner [95]. CD226 deficiency or blocking with anti-CD226 mAbs rendered tumor-bearing mice resistant to the combined treatment, which implies that CD226 is required for the efficacy of anti-PD-1 and anti-GITR mAb combination treatment. In a mouse melanoma model, CD8^+^TILs showed differential responsiveness to anti-PD-1 mAb treatment by CD226 expression [100].

## 4. Conclusions

TIGIT has emerged as a promising target for next generation cancer immunotherapy. Several clinical trials are currently evaluating the efficacy of anti-TIGIT mAbs in patients with different types of cancer. The most advanced candidate, tiragolumab, has exhibited remarkable efficacy in PD-L1-positive NSCLC patients in phase II clinical trials, in combination with PD-L1 blockade. However, the mode of action of TIGIT blockade remains to be fully elucidated. First, the association of CD226 activation with the efficacy of TIGIT blockade needs to be confirmed in clinical trials. Importantly, CD226^hi^CD8^+^T cells representing a predictive biomarker for several ICB therapies in a large cohort of patients with different cancer types need to be examined. Second, whether Fc engagement is necessary and required for the anti-tumor efficacy of TIGIT blockade remains to be defined. Further, it remains unclear whether the therapeutic effect of anti-TIGIT antagonist is mediated by the reactivation of T_ex_ or NK cells, the depletion of T_reg_ cells, the reprogramming of APC functions, or all of them. Several ongoing clinical trials will likely help provide answers to these questions.

## Figures and Tables

**Table 1:** Clinical trials of TIGIT inhibitors.

TIGIT Inhibitor	Sponsor	Isotype	Identifiers	Cancer Type	Combination	Phase	Recruitment Status	Start Date
ASP-8374	Astellas Pharma Inc.	IgG4	NCT03260322	Advanced solid tumor	ASP-8374 alone; Pembrolizumab (anti-PD-1)	Phase 1b	No longer recruiting	8 September 2017
			NCT03945253	Advanced solid tumor	ASP-8374 alone	Phase 1	No longer recruiting	5 August 2019
BGB-A1217	BeiGene Co Ltd.	IgG1	NCT04047862	Advanced solid tumor	Tislelizumab (anti-PD-1)	Phase 1	Recruiting	26 August 2019
BMS-986207	Bristol-Myers Squibb Co.	IgG1 (Fc receptor disabled)	NCT02913313	Advanced solid tumor	BMS-986207 alone; Nivolumab (anti-PD-1)	Phase 1/2	No longer recruiting	29 November 2016
			NCT04150965	Multiple myeloma	BMS-986207 alone; Dexamethasone+Pomalidomide	Phase 1/2	Recruiting	16 April 2018
			NCT04570839	Advanced solid tumor	COM-701 (PVRIG inhibitor) + Nivolumab (anti-PD-1)	Phase 1/2	Recruiting	31 August 2020
			NCT04065425	Multiple myeloma	Dexamethasone + Pomalidomide	Phase 1/2	Not yet recruiting	1 October 2019
COM-902	Compugen Ltd.	IgG4	NCT04354246	Advanced solid tumor	COM-902 alone	Phase 1	Recruiting	31 March 2020
AB154 (Domvanalimab)	Arcus Biosciences Inc.	IgG1 (Fc receptor disabled)	NCT03628677	Advanced malignancy	AB154 alone; Zimberelimab (anti-PD-1)	Phase 1	Recruiting	12 September 2018
			NCT04656535	Recurrent Glioblastoma	Zimberelimab (anti-PD-1)	Phase 1	Not yet recruiting	31 January 2021
			NCT04262856	PD-L1 positive lung cancer	Zimberelimab (anti-PD-1); Zimberelimab + etrumadenant (A2aR and A2bR antagonist)	Phase 2	Recruiting	28 May 2020
EOS-884448	iTeos Therapeutics	IgG1	NCT04335253	Advanced tumor	EOS-884448 alone	Phase 1/2	Recruiting	18 February 2020
Etigilimab (OMP-313M32)	OncoMed	IgG1	NCT03119428	Advanced solid tumor	Etigilimab alone; Nivolumab (anti-PD-1)	Phase 1	Terminated	2 May 2017
IBI-939	Innovent Biologics Inc.	Not disclosed	NCT04353830	Advanced tumor	IBI-939 alone; Sintilimab (anti-PD-1)	Phase 1a	Recruiting	22 May 2020
			NCT04672356	Advanced lung cancer	Sintilimab (anti-PD-1)	Phase 1a	Not yet recruiting	28 January 2021
			NCT04672369	Advanced NSCLC	Sintilimab (anti-PD-1)	Phase 1b	Not yet recruiting	6 June 2021
M-6223	Serono Research Institute Inc, Merck KGaA	Not disclosed	NCT04457778	Advanced solid tumor	M-6223 alone; Bintrafusp alfa (TGF beta ligand inhibitor)	Phase 1	Recruiting	10 July 2020
Vibostolimab (MK-7684)	Merck Sharp & Dohme Corp.	IgG1	NCT02964013	Advanced solid tumor	Vibostolimab alone; Pembrolizumab (anti-PD-1); Pembrolizumab + Pemetrexed + Carboplatin; Pembrolizumab + Carboplatin or Cisplatin + Etoposide	Phase 1	Recruiting	13 December 2016
			NCT04305054	Advanced melanoma	Pembrolizumab (anti-PD-1);	Phase 1/2	Recruiting	1 July 2020
			NCT04303169	Melanoma	Pembrolizumab (anti-PD-1)	Phase 1/2	Recruiting	26 June 2020
			NCT04305041	Refractory melanoma	Pembrolizumab + Quavonlimab (anti-CTLA4)	Phase 1/2	Recruiting	26 June 2020
			NCT04165070	Advanced NSCLC	Pembrolizumab + Carboplatin + Paclitaxel; Pembrolizumab + Pemetrexed	Phase 2	Recruiting	19 December 2019
			NCT02861573	Prostate cancer	Pembrolizumab (anti-PD-1)	Phase 1/2	Recruiting	17 November 2016
Tiragolumab (MTIG7192A)	Genentech Inc., Chugai Pharmaceutical Co. Ltd., Roche Holding AG	IgG1	NCT04045028	Relapse/Refractory Multiple myeloma and B-cell Non-Hodgkin lymphoma	Tiragolumab alone; Daratumumab (anti-CD38); Rituximab (anti-CD20)	Phase 1	Recruiting	22 July 2019
			NCT02794571	Metastatic solid tumor	Tiragolumab alone; Atezolizumab (anti-PD-L1); Chemotherapy (Carboplatin, Cisplatin, Etoposide, Paclitaxel, Pemetrexed)	Phase 1	Recruiting	23 May 2016
			NCT03281369	Metastatic esophageal cancer	Atezolizumab (anti-PD-L1); Atezolizumab + Cisplatin+5FU	Phase 1/2	Recruiting	13 October 2017
			NCT04513925	NSCLC	Atezolizumab (anti-PD-L1)	Phase 3	Recruiting	24 August 2020
			NCT04294810	Metastatic NSCLC, PD-L1 selected	Atezolizumab (anti-PD-L1)	Phase 3	Recruiting	04 March 2020
			NCT04665843	Metastatic head and neck cancer, PD-L1 positive	Atezolizumab (anti-PD-L1)	Phase 2	Not yet recruiting	21 January 2021
			NCT04543617	Esophagus squamous cell carcinoma	Atezolizumab (anti-PD-L1)	Phase 3	Recruiting	28 September 2020
			NCT04300647	Metastasis/Recurrent uterine cervix tumor, PD-L1 positive	Atezolizumab (anti-PD-L1)	Phase 2	Recruiting	30 June2020
			NCT03563716	NSCLC, chemotherapy-naïve	Atezolizumab (anti-PD-L1)	Phase 2	No longer recruiting	10 August 2018
			NCT04665856	Small-cell lung cancer	Atezolizumab + Carboplatin + Etoposide	Phase 3	Recruiting	4 January 2021
			NCT04619797	Metastatic NSCLC	Atezolizumab + Pemetrexed + Carboplatin or Cisplatin	Phase 2	Recruiting	11 December 2020
			NCT04584112	Triple-negative breast cancer	Atezolizumab + Nab-paclitaxel; Atezolizumab + Nab-pac-carbo-AC; Atezolizumab+Nab-pac-AC;	Phase 1b	Recruiting	28 September 2020
			NCT04256421	Metastatic small-cell lung cancer	Atezolizumab + Carboplatin + Etoposide	Phase 3	Recruiting	4 February 2020
			NCT04540211	Metastatic esophageal cancer	Atezolizumab + Paclitaxel + Cisplatin	Phase 3	Recruiting	4 November 2020
			NCT04524871	Metastatic hepatocellular carcinoma	Atezolizumab + Bevacizumab (anti-VEGF)	Phase 1/2	Recruiting	2 November 2020
			NCT03869190	Advanced urothelial carcinoma	Atezolizumab (anti-PD-L1)	Phase 1/2	Recruiting	1 June 2019
			NCT03193190	Metastatic pancreatic ductal adenocarcinoma	Atezolizumab + Nab-Paclitaxe l+ Gemcitabine	Phase 1/2	Recruiting	5 July 2017

PD-L1: programmed death-ligand 1; NSCLC: non-small cell lung cancer; TGF: transforming growth factor; VEGF: vascular endothelial growth factor.
